# Effectiveness of clinical decision support in controlling inappropriate red blood cell and platelet transfusions, speciality specific responses and behavioural change

**DOI:** 10.1186/s12911-022-02045-8

**Published:** 2022-12-29

**Authors:** Jolene Atia, Felicity Evison, Suzy Gallier, Sophie Pettler, Mark Garrick, Simon Ball, Will Lester, Suzanne Morton, Jamie Coleman, Tanya Pankhurst

**Affiliations:** 1 University Hospital Birmingham NHS Foundation Trust, Edgbaston, Birmingham, B15 2GW UK; 2 PIONEER: HDR-UK Health Data Research Hub for Acute Care, Institute of Inflammation and Ageing, University of Birmingham, Birmingham, B15 2GW UK; 3 School of Medicine, College of Medical and Dental Sciences, University of Birmingham, Edgbaston, Birmingham, B15 2TT UK; 4 HDRUK Better Care Science Priority and Health Data Research UK Midlands, University of Birmingham, Birmingham, UK; 5 NHS Blood and Transplant, Vincent Drive, Edgbaston, Birmingham, B15 2SG UK; 6 Department of Health Informatics, University Hospital Birmingham NHS Foundation Trust, Edgbaston, Birmingham, B15 2GW UK

**Keywords:** Red blood cells, Platelets, Haemoglobin, Transfusion, Clinical decision support, CDS, Electronic health records, EHR, e-Alerts, Segmented linear regression of interrupted time series

## Abstract

**Background:**

Electronic clinical decision support (CDS) within Electronic Health Records has been used to improve patient safety, including reducing unnecessary blood product transfusions. We assessed the effectiveness of CDS in controlling inappropriate red blood cell (RBC) and platelet transfusion in a large acute hospital and how speciality specific behaviours changed in response.

**Methods:**

We used segmented linear regression of interrupted time series models to analyse the instantaneous and long term effect of introducing blood product electronic warnings to prescribers. We studied the impact on transfusions for patients in critical care (CC), haematology/oncology (HO) and elsewhere.

**Results:**

In non-CC or HO, there was significant and sustained decrease in the numbers of RBC transfusions after introduction of alerts. In CC the alerts reduced transfusions but this was not sustained, and in HO there was no impact on RBC transfusion. For platelet transfusions outside of CC and HO, the introduction of alerts stopped a rising trend of administration of platelets above recommended targets. In CC, alerts reduced platelet transfusions, but in HO alerts had little impact on clinician prescribing.

**Conclusion:**

The findings suggest that CDS can result in immediate change in user behaviour which is more obvious outside specialist settings of CC and HO. It is important that this is then sustained. In CC and HO, blood transfusion practices differ. CDS thus needs to take specific circumstances into account. In this case there are acceptable reasons to transfuse outside of these crude targets and CDS should take these into account.

**Supplementary Information:**

The online version contains supplementary material available at 10.1186/s12911-022-02045-8.

## Background

Blood component transfusion is used widely in healthcare and is life-saving in many clinical settings such as trauma, critical care (CC) and haemato-oncology (HO). The transfusion of blood components has known risks of harm if used unnecessarily. Serious short-term adverse effects of red blood cells (RBCs) include transfusion related circulatory overload, allergic reactions, acute haemolytic transfusion reactions, and transfusion related acute lung injury [1]. Blood transfusions rely on limited resources and are expensive to collect, store, distribute and administer. A unit of RBCs and its associated administration costs £139 and £58, respectively [2].

Patient blood management is the international, multidisciplinary, evidence-based approach to optimising the care of patients who may need a blood transfusion. Patients with asymptomatic anaemia alone do not necessarily achieve improved outcomes following transfusion [3] and furthermore those patients should be investigated to treat the cause of anaemia. Most recent evidence has shown that restrictive blood transfusion strategies are as safe as liberal transfusion strategies and are feasible in many populations [4, 5]. As a result, it is important that only patients who have an indication for transfusion and for whom a suitable safer alternative is not available should be prescribed blood components. In the UK, National Institute for Health and Care Excellent (NICE) guidelines [6] inform management of blood transfusion based on best available evidence.

In order to ensure the delivery of appropriate clinical care with regards to the need for transfusion, a number of strategies have been implemented in clinical practice. These often include a reliance on educational interventions including promoting or enforcing clinical guidance, providing senior oversight or requiring prior authorisation by clinical or laboratory staff, and monitoring use [7].

In hospitals using Electronic Health Records (EHRs) with Computerised Provider Order Entry, decision making can be supported by patient specific assessments or recommendations as warnings or alerts. This clinical decision support (CDS) demonstrably improves quality of care and patient outcomes preventing prescribing errors by provision of immediate access to relevant information such as the presence of a duplicate drug, a drug disease contraindication, or inappropriate drug dosing [8]. When there is linkage of laboratory data within the EHR, then laboratory results can be used to drive CDS warnings, for example, to guide medication dosing in renal insufficiency [9].CDS can not only be used to guide best practice but has also been used to improve appropriate utilisation of precious healthcare resources.

More recently, CDS has also been investigated as a way to improve the prescription of blood components and targeted CDS has been shown to promote restrictive RBC transfusion practice [10, 11]. The overall aim of this retrospective cohort study is to assess the effects of specific CDS warnings triggered when prescribing blood components in the UK and clinician behavioural changes in response to these. Warnings were based on existing laboratory data that might suggest inappropriate RBC and platelet transfusion. Acceptability of CDS to clinicians is important in adoption. Where clinicians do not feel CDS is clinically relevant it will often be boycotted.

## Methods

### Setting and context

This study was conducted in a large, urban teaching hospital within the UK, that has an in-house built clinically-led EHR; PICS (Prescribing, Information and Communication System) which has been described elsewhere [12, 13]. Doctors, nurses and allied health professionals co-design the content of the EHR, working directly with programmers to produce software that is functional for the end-user. The hospital is a major centre for trauma, cardiac surgery, solid organ transplantation and haematology and oncology, and has a large 80 bedded intensive care unit. There is no paediatric or obstetric care. PICS provides users with CDS, based on user privilege, clinical protocols and best practice guidelines. Alerts/advice appear to the prescriber as orders are tiered in severity and can be interruptive or non-interruptive. This allows alerts to be tailored, for example, as opposed to blanket alerting that appears in some EHR.

### Description of the intervention

The PICS EHR is implemented throughout the hospital, and is continuously updated to incorporate rules and guidance to improve patient safety, efficiency and best practice. Senior haematologists requested the implementation of CDS warnings to reduce inappropriate transfusion of RBCs and platelets.

Three changes to the EHR were made in respect RBC transfusion:

1. May 2012: a warning was shown when clinicians prescribed RBC for patients who had a haemoglobin ≥ 100 g/L. Users were asked to consider if transfusion was appropriate.
2. May 2015: (in addition to the first warning): a further warning appeared in non-CC patients if haemoglobin was ≥ 80 g/L and in CC if haemoglobin was ≥ 70 g/L in response to prescription of RBCs. The warning provided details of the most recent haemoglobin level and its date.
3. July 2016: (replacing the warning introduced in May 2015). A warning was triggered for all patients if haemoglobin was ≥ 70 g/L in all clinical locations.

Similar changes were made in respect of platelet transfusion:

1. May 2015: a warning was shown when clinicians prescribed platelets if the platelet count was > 20 × 10^9^/L in all locations. Users were asked to consider if platelet transfusion was appropriate.
2. July 2016: the limit was lowered so that the warning appeared if the platelet count was > 10 × 10^9^/L.

**Fig. 1:**
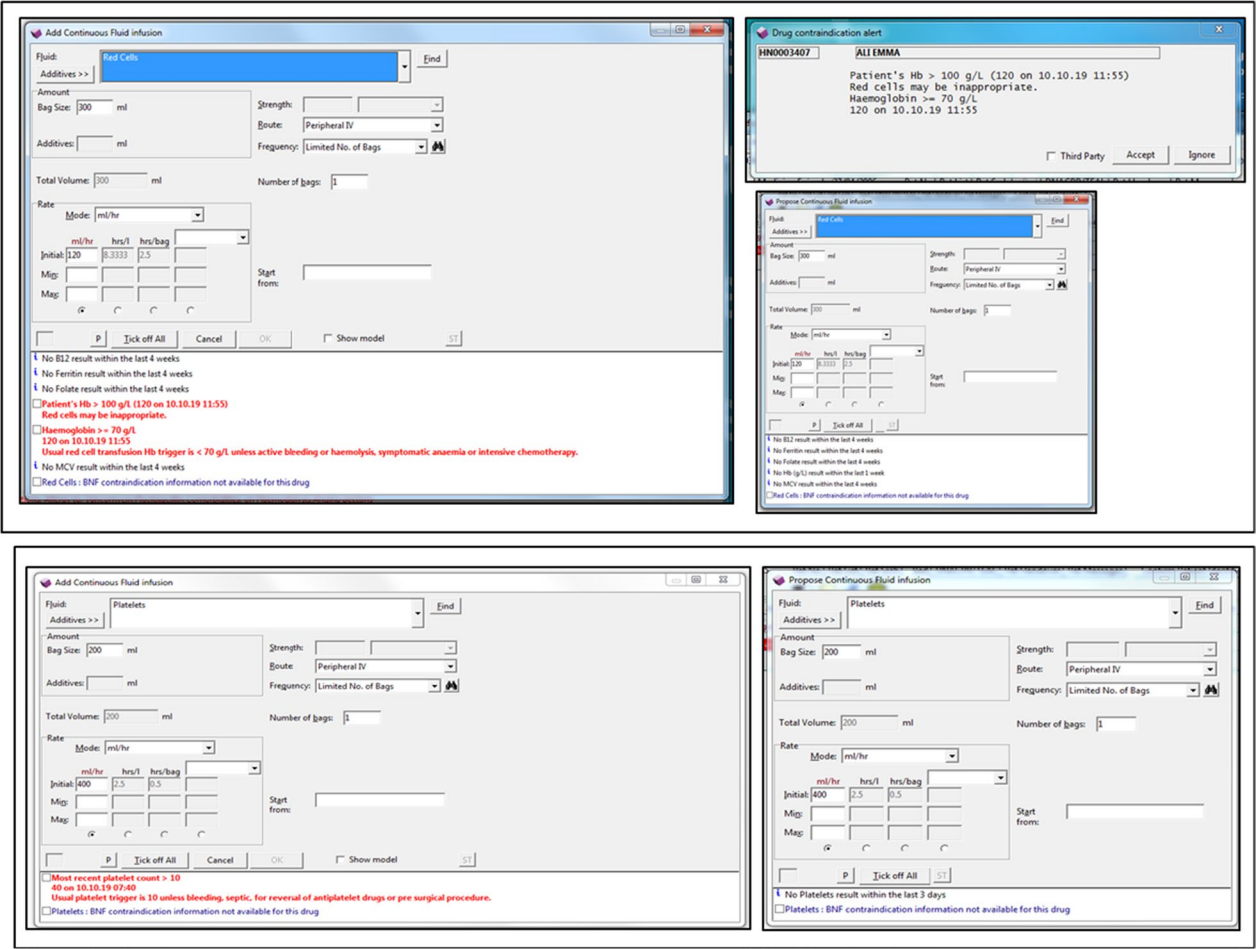
Screenshots of the different RBC (top) and platelets (bottom) transfusion warnings currently in place in the Electronic Health Record, PICS

Warnings were evoked when a RBC transfusion was prescribed, based on a haemoglobin result (available in the 7 days prior to the prescription attempt), while the platelets warning was triggered if a platelet count was available within the preceding 3 days (Fig. 1). Both required the user to enter their password in order to proceed with the prescription. If neither results for haemoglobin within the past 7 days nor platelet count within 3 days existed, a warning that did not require user acknowledgment was displayed in the prescription window.

### Study population

Data were extracted for all patients who were prescribed RBC transfusion between January 2010 and August 2019. We excluded patients on a renal dialysis programme and patients in emergency department. This was pragmatic as the electronic health record is not used for prescribing and administration in the dialysis units where much of the administration is undertaken. Patients under the care of HO and patients in CC were investigated separately since clinician behaviour in relation to transfusion in these specialties is often different to the general hospital population within our institution.

Data on each patients’ haemoglobin results in the 7 days prior to the prescription, if available, were also extracted. For analysis the monthly numbers of RBC transfusion prescriptions for patients with preceding haemoglobin results ≥ 70 g/L, 80 g/L, 100 g/L were calculated.

Similarly, data on all patients with a prescription for a platelet transfusion were obtained for the period from January 2010 to August 2019 with the patients’ platelets results within 3 days prior to the transfusion prescription, if available. For the analysis, the total monthly numbers of prescriptions for a platelet transfusion was calculated as well as the monthly number of prescriptions for a platelet transfusion where the patient had a platelets count > 10 × 10^9^/L within the 3 days prior. To reflect the clinical practice using the EHR, missing data was assumed not to have been measured and not included in the denominator or numerator.

### Statistical methods

The effect of the various warnings was analysed using segmented linear regression of interrupted time series models [14]. These models looked at the changes in number of patients per month who had a prescription for a transfusion before and after the introduction of the warnings. Monthly data for three cohorts of patients who had RBC transfusion with haemoglobin ≥ 70 g/L (as a percentage of the total number of all RBC transfusions in this cohort) was collated: patients not in CC or under the care of HO (non-CC/HO), those on CC, and those under HO. For each dataset, seven variables were generated to define the time each intervention was introduced and the period of time for which it was in place (Additional file 1: Table S1). A numeric variable identifying the time period (in month) of the study was introduced; the coefficient for this variable reflected the change of the outcome with time in the pre-intervention period. Three similar variables were created that took the value of zero pre-interventions and the month number since the introduction until the override of the intervention. The alert for haemoglobin ≥ 100 g/L did not have an override. To consider the step change in the outcome due to the three interventions, three additional binary variables, with value of zero before the intervention and one after the intervention, were generated.

A similar approach was used for the platelet-related warnings. In this case the model included five variables to account for the changes in level and trend of the two interventions and the extra variable comprising the months’ number since the start until the end of the study period (Additional file 1: Table S2). The analysis was performed with the three cohorts who had platelet transfusion with platelet count > 10 × 10^9^/L. Significant results were reported at p < 0.05. All analyses were undertaken in R (R Core team, 2020) and graphs were generated using ggplot2 package version 3.2.1 [15].

## Results

### RBC transfusion

**Fig. 2:**
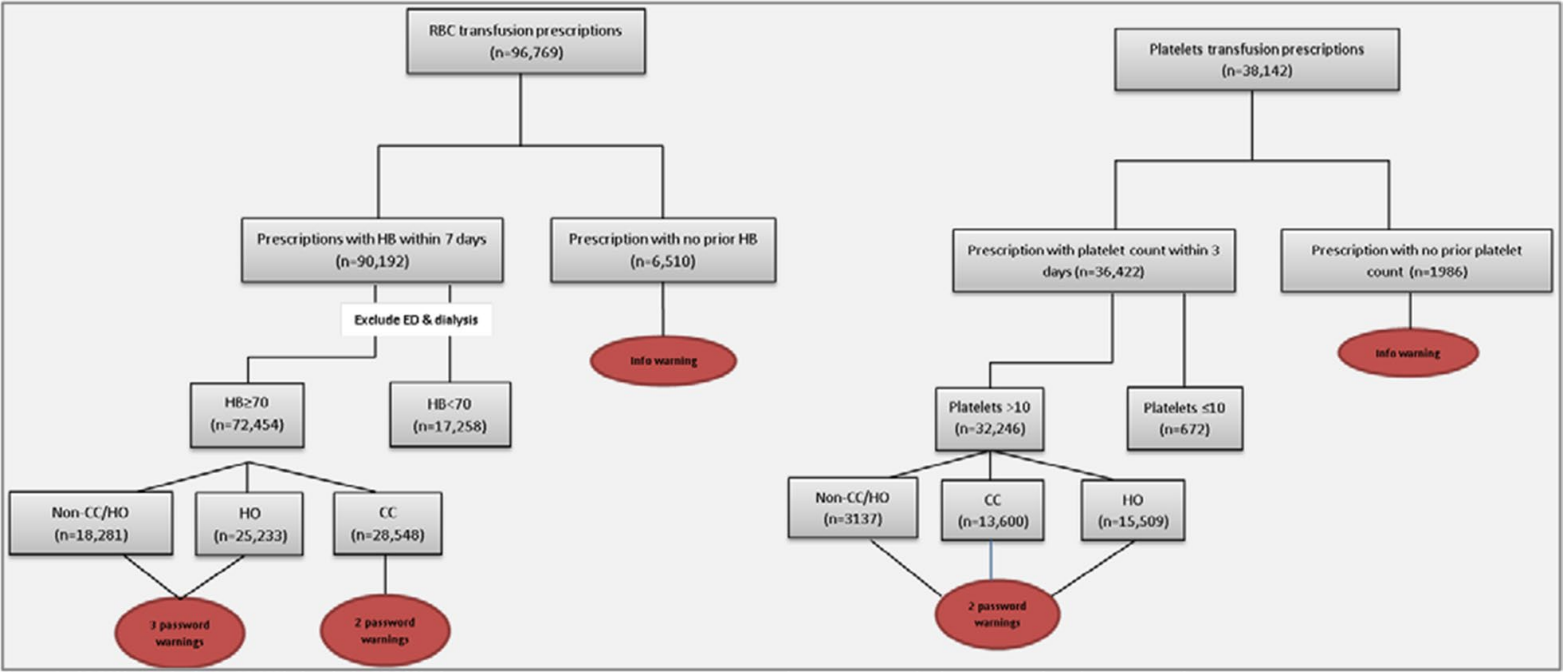
Flow diagram of the extracted data with the warnings for each category for the red blood cell (RBC) transfusion prescription (left) and the platelets transfusion prescription (right). HB: haemoglobin; ED: emergency department; CC: critical care; HO: haematology or oncology. Many transfusions were prescribed without haemoglobin or platelet results being available; prior to all point of care analysers being networked with PICS some RBC results would not have been available for extraction, or decisions to transfuse may have been based on blood gas results which were not extracted. In these cases prescribers would be presented with advice messages but warnings triggered by results would not be available. Password warnings for RBC transfusion (1) May 2012: a warning was shown when clinicians prescribed RBC for patients who had a haemoglobin ≥ 100 g/L. (2) May 2015: (in addition to the first warning): a further warning appeared in non-CC/HO patients if haemoglobin was ≥ 80 g/L and in CC if haemoglobin was ≥ 70 g/L in response to prescription of RBCs. (3) July 2016: (replacing the warning introduced in May 2015). A warning was triggered for all patients if haemoglobin was ≥ 70 g/L in all clinical locations. Warnings for platelet transfusions: (1) May 2015: a warning was shown when clinicians prescribed platelets if the platelet count was > 20 × 10^9^/L in all locations. (2) July 2016: the limit was lowered so that the warning appeared if the platelet count was > 10 × 10^9^/L

**Fig. 3:**
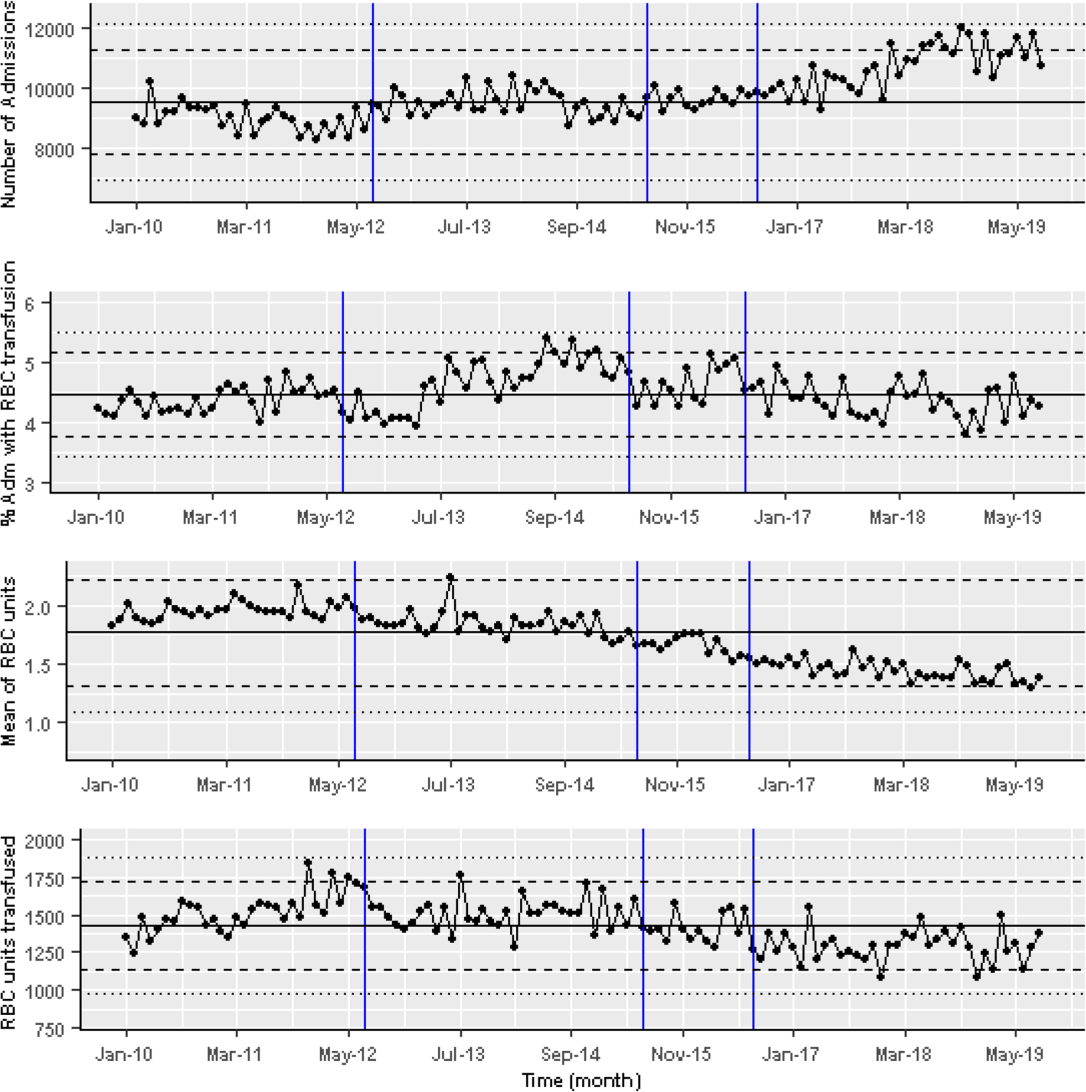
From top to bottom, monthly number of admissions to the hospital, percentage of patients who receive an RBC transfusion, mean number of monthly RBC units transfused in a transfusion episode, and total monthly number of RBC units transfused in the hospital

There were a total of 96,769 RBC transfusion prescriptions in the hospital during the study period. These prescriptions were ordered in 72,545 patients with haemoglobin greater than or equal to 70 g/L; 18,281 (25.2%) were prescribed to non-CC/HO patients, 28,548 (39.4%) to patients in CC, and 25,233 (34.8%) to HO patients (Fig. 2). The average of the monthly percentage of prescriptions with haemoglobin ≥ 70 g/L throughout the whole study period were 60.63%, 84.62%, and 81.2% for non-CC/HO patients, CC, and HO patients respectively (median shown in Fig. 3, black line). Overall, during the study period the percentage of patients receiving a RBC transfusion in the trust remained fairly stable, however the number of units of blood that patients received fell (Fig. 3). The absolute number of RBC units transfused and the mean number of monthly RBC units transfused in a transfusion episode dropped slightly despite the number of admissions rising (Fig. 3).

The median haemoglobin for the patients who were prescribed RBC transfusion within 7 days was 74 (interquartile range, IQR 67–81), 79 (IQR 73–92), and 81 (IQR 75–88) for the non-CC/HO, CC, and HO patients, respectively.

**Fig. 4:**
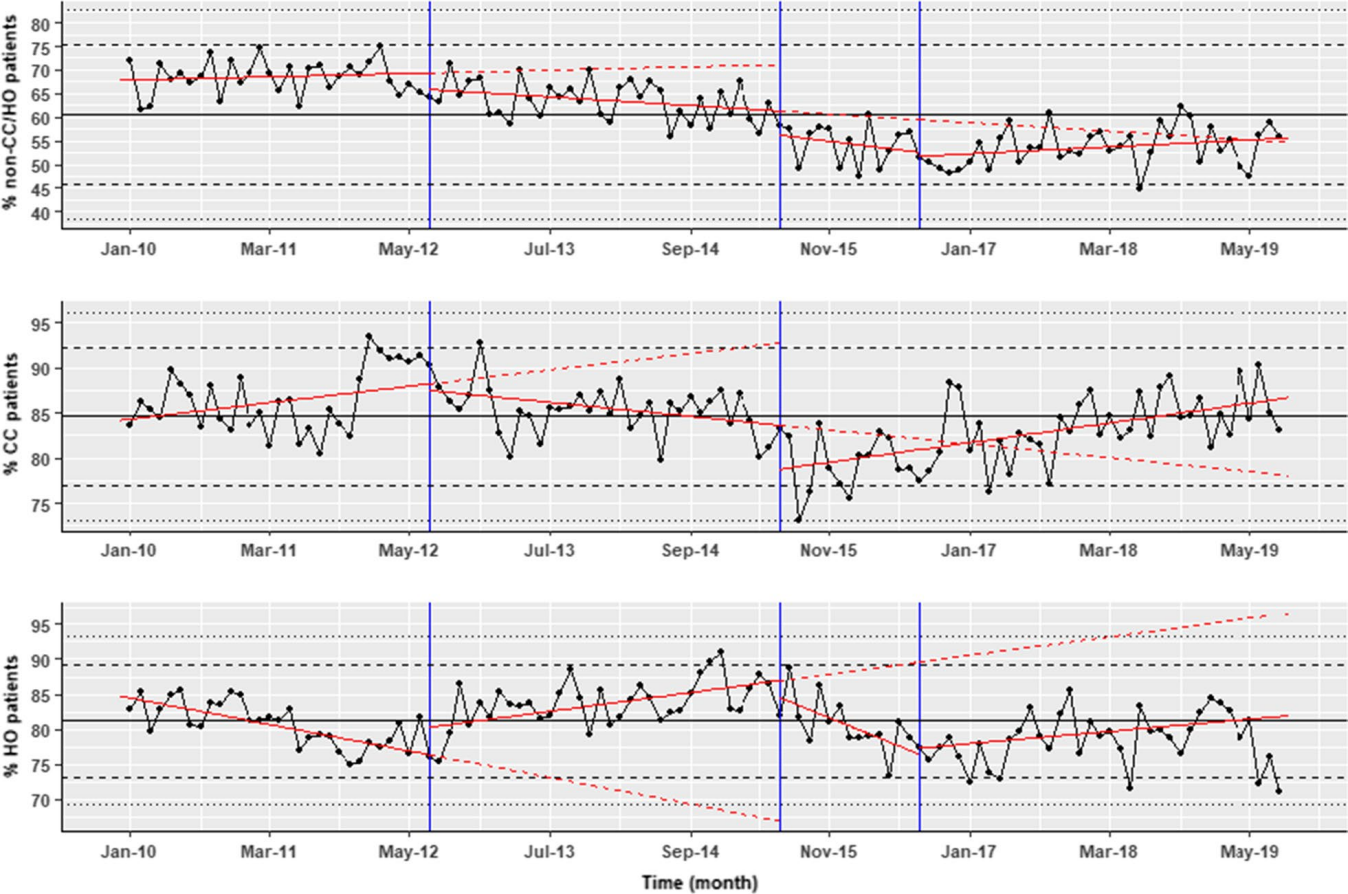
Time series of the number of RBC transfusion prescription to non-CC/HO patients (upper panel), CC (middle panel) and HO (lower panel) patients with haemoglobin ≥ 70 g/L as a percentage of the total transfusion in the Trust in those cohorts. Also shown the cumulative model fit for each segment (red line), the intervention time (blue), the trend lines prior to the interventions being introduced (dashed red lines), median of the whole data (black), two and three standard deviation from the median (dashed and dotted black lines, respectively)

For the cohort of non-CC/HO patients, our analysis found a significant drop in the monthly proportion of patients prescribed RBCs following the third change made to the warning (coefficient − 7.25, 95% CI − 11.5 to − 3.5; p < 0.01) from the trend prior to this warning. The initial two warnings did not have a significant effect, although there is weak evidence of a step after the introduction of the first and second warnings, and a reduction in the gradient after the first warning was introduced, compared to the fitted line prior to this warning being introduced. The models’ coefficients are shown in Table 1. Lines representing the linear regression fit are included to show the cumulative effects of the trend, accounting for the changes from the previous trend following the interventions (Fig. 4). Overall, after the introduction of all the warnings in the EHR there is a reduction in transfusion with prior haemoglobin ≥ 70 g/L for this cohort. Before the introduction of all the warnings, on average 65% of transfusions were given to patients with a prior haemoglobin ≥ 70 g/L but at the end of the study this had fallen to 55% (Fig. 4 top panel).

**Table 1:** Linear regression for RBC transfusion for patients with HB70 ≥ g/L

	Non-CC/HO		CC		HO	
	Coefficient	*p* value	Coefficient	*p* value	Coefficient	*p* value
Intercept	67.86	< 0.001*	84.19	< 0.001*	84.78	< 0.001*
Gradient pre warnings	0.05	0.56	0.13	0.054	− 0.27	< 0.001*
Step change post warning 1	− 3.29	0.09	− 0.48	0.76	3.45	0.018*
Change in gradient post warning 1	− 0.18	0.08	− 0.24	0.005*	0.46	< 0.001*
Step change post warning 2	− 4.98	0.06	− 5.12	< 0.001*	− 1.63	0.4
Change in gradient post warning 2	− 0.13	0.65	0.26	< 0.001*	− 0.77	< 0.001*
Step change post warning 3	− 7.52	0.002*	–	–	− 12.26	< 0.001*
Change in gradient post warning 3	0.22	0.016*	–	-	− 0.07	0.323

*Significant at p < 0.05

For CC patients, there was a significant small linear decrease in gradient compared to the original fitted line, after the introduction of the first warning (p < 0.01). Following the second intervention, a significant large step-decrease to the prior trend was observed as soon as the warning was introduced (coefficient − 5.12, p < 0.001; 95% CI − 7.978 to − 2.27), then the gradient started to change, increasing significantly, with coefficient 0.26 (p < 0.001; 95% CI 0.13–0.39), until the end of the study period.

For HO patients, changes in the trend and the step level were evident throughout the study period. The first warning was followed by a large step increase and a positive change to the initial gradient. After the second warning, although no significant step-change in transfusion was observed (p = 0.4), there was an associated significant linear decrease (p < 0.001) to the previously fitted gradient. There was a further significant drop and a linear increase to the trend line after the third warning (p < 0.001). Warnings have reduced the overall rise in RBC prescription throughout the study period but the number of potentially inappropriate transfusions is not significantly lower by the end of the study.

### Platelet transfusion

**Fig. 5:**
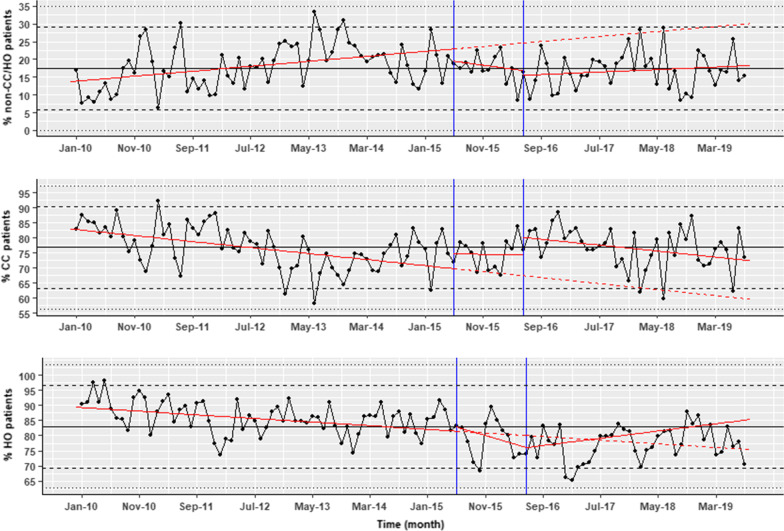
Time series of the number of platelets transfusion prescriptions to non-CC/HO patients (upper panel), CC (middle panel), and HO (lower panel) patients with platelets > 10X10^9^/L as a percentage of the total transfusion in those cohorts. Also shown the cumulative results from the linear regression model fit for each segment (red line), the intervention time (blue), the trend lines prior to the interventions being introduced (dashed red lines), median of the whole data (black), two and three standard deviation from the median (dashed and dotted black lines, respectively)

**Fig. 6:**
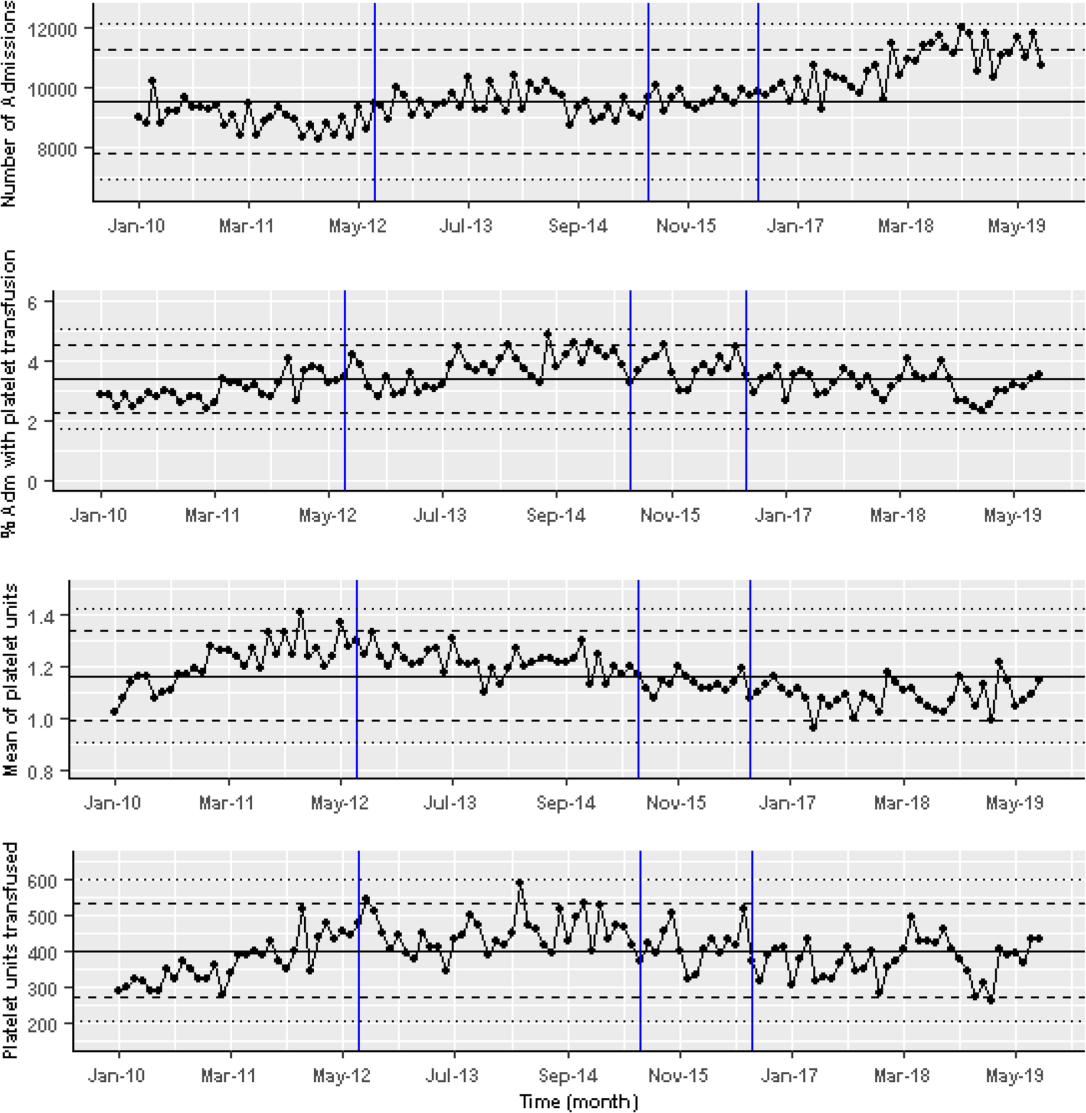
From top to bottom, monthly number of admissions to the hospital, percentage monthly admissions with platelet transfusion, mean number of monthly platelet units transfused in a transfusion episode, and absolute monthly number of platelet units transfused

There were 38,142 platelets transfusion prescriptions in the hospital during the study. 3137 (8.22%) prescriptions were for non-CC/HO patients, 13,600 (35.66%) for CC patients, and 15,509 (40.66%) for HO patients. The monthly percentages of those who had transfusion in the 3 days prior with a platelets result > 10 × 10^9^/L are shown in Fig. 5, together with linear regression model fit showing the cumulative changes in the trend and level over time. The model coefficients are reported in Table 1. On average, platelets were transfused where the platelet count was > 10 × 10^9^/L in 17.5%, 76.8%, 83% in the non-CC/HO, CC and HO cohorts, respectively (median shown in Fig. 5, black line). The median 3 days pre-transfusion platelet counts in these groups were 58 (IQR 36–102), 71 (IQR 39–115), and 24 (IQR 17–38), respectively. During the study period the percentage of patients receiving platelet transfusion in the trust remained fairly stable; however the number of units of platelets that patients received fell slightly. This is shown in Fig. 6 where the absolute number of platelet units transfused and the mean number of monthly platelet units transfused in a transfusion episode dropped slightly despite the number of admissions rising.

In the cohort of non-CC/HO patients, there was a small linear upward trend in the monthly proportion of platelet transfusions given with a prior platelet count > 10 × 10^9^/L throughout the entire study period (p < 0.001). There was no evidence of a change in the level or the gradient after the introduction of the first warning (Fig. 5, Table 2), nor in the gradient after the change made in the warning. After the second change in the warning, a significant step-decrease from the trend prior to this warning was observed (p < 0.001). At the end of the study however this change was not sustained and overall the proportion of platelet transfusions given with a pre-transfusion platelet count > 10 × 10^9^/L had not changed.

**Table 2:** Linear regression for platelets transfusion for patients with platelets > 10 × 10^9^/L

	Non-CC/HO		CC		HO	
	Coefficient	*p* value	Coefficient	*p* value	Coefficient	*p* value
Intercept	13.75	< 0.001*	83.1	< 0.001*	89.45	< 0.001*
Gradient pre warnings	0.14	< 0.001*	− 0.2	< 0.001*	− 0.1	0.004*
Step change post warning 1	− 3.49	0.295	4.87	0.199	1.34	0.675
Change in gradient post warning 1	− 0.36	0.279	0.16	0.66	− 0.44	0.173
Step change post warning 2	− 8.76	0.001*	12.78	< 0.001*	− 5.61	0.03*
Change in gradient post warning 2	− 0.1	0.274	0.002	0.981	0.22	0.015*

*Significant at p < 0.05

In the CC patients there was a small linear decrease (p < 0.001) in the proportion of patients transfused throughout the whole study. No evidence was found of a change in the level or the gradient after the first intervention, nor in the gradient after the second intervention. In contrast to the non-CC/HO cohort, a significant step-increase from the trend prior to this intervention was recorded following the second intervention (p < 0.001).

For HO patients, there was a small linear decrease in the monthly proportion of prescriptions overall (p = 0.004). After the first warning, there was no significant change in the gradient or level. After the second warning a significant linear increase and a significant drop compared to the trend prior to the second intervention was also observed (p = 0.03). The results of the analysis are shown in Table 2.

## Discussion

The literature on whether warnings and interruptive alerts in EHRs positively impacts patient care is mixed. A small, non-significant effect on RBC transfusion has been demonstrated in a previous study [16], while other studies revealed a significant positive effect, especially on RBCs [17–22], while others reported a non-significant decrease in inappropriate fresh frozen plasma and platelets transfusion [21, 23]. Goodnough et al. [24] reported an improvement in many outcomes after introducing blood component-related CDS rules. These include a decline in RBC utilisation, a decrease in mortality, length of hospital stay, 30-days readmission rate, and in the total hospital transfusion-related costs of RBCs. A randomised control trial of a CDS intervention has demonstrated effectiveness of CDS involving all blood components [25].

In line with national and international guidelines as supported by evidence from clinical trials, the haematologists at our institution requested changes to the EHR to educate users and attempt to reduce inappropriate transfusions, as implied by these guidelines. Guidelines of course cannot infer that all transfusions out with them are inappropriate but it is likely that a large proportion would be. Initial levels for inappropriate transfusions were lowered over time reflecting national guidance [6]. We were also very interested in studying the large groups of patients in CC and HO as distinct groups for two reasons, firstly these patients receive a significant proportion of RBC and platelet transfusions in the trust (74.23% of RBC transfusion and 90.27% of platelets transfusion) and secondly, prescribing behaviour is likely to be different in these groups for reasons discussed below. Of note 8% of platelet transfusions were given to patients outside of critical care or haematology/oncology. Although many patients will be specialty specific, some patients with chronic haematological conditions or cancers will be cared for by other specialities and therefore be spread across the hospital.

There are no studies that help answer the question about why different specialties react differently to warnings, and this is perhaps one of the most interesting outcomes. One could imagine that haematologists are unlikely to think that they need automated prompts to inform their transfusion decisions, and junior doctors in these areas may defer to senior colleagues. In ICU more exceptions are made to usual practice in our experience. This is a relevant area of research as ‘one size fits all’ for CDS may need to be nuanced across hospital specialties. We continuously update and modify the EHR based on new evidence, user feedback and behaviours.

There are important reasons why RBC and platelet transfusions may be appropriate above the warning thresholds set in the EHR. Platelets may be needed in active bleeding and prior to invasive procedures. The Trust is a major trauma centre, frequently treating patients with major haemorrhage.

We found that the warnings did change user behaviour. The effect was most obvious outside the specialist settings of CC and HO, where there was a significant reduction in RBC transfusions given to patients with haemoglobin of over 70 g/L. While it has been longstanding practice to transfuse RBC at somewhat arbitrary thresholds, evidence from large randomised controlled trials over the last 20 years supports use of restrictive thresholds [26].

Blood transfusion can be utilised differently in HO where many patients will have chronic, transfusion dependent anaemia and be transfused to a personalised threshold depending on their symptoms. In oncology, patients are frequently transfused to a haemoglobin > 100 g/L prior to starting chemotherapy, although this practice is not evidence based and contravenes local and national guidance. Due to the lack of evidence to support use of restrictive thresholds for RBC transfusion in patients undergoing intensive treatment for haematological malignancy, trust guidelines recommended a threshold of 80 g/L in this group until 2019.

In CC we did not see a significant effect on transfusion after introducing warnings. In fact the feedback from clinicians in this area was negative in respect to these warnings, and we were repeatedly asked to remove them. Utilising questionnaires and focus groups users stated that this warning contributed to alert fatigue, and was perceived as non-contributory to patient care. In some situations, such as acute coronary syndrome, guidelines exclude the 70 g/L threshold, which may influence prescribing in cardiac CC [6]. Furthermore, there are randomised controlled trials in patients undergoing cardiac surgery which largely support the safety of restrictive thresholds but the outcomes of which are subject to debate [27, 28].

This contributes importantly to understanding how different areas will react to CDS dependent on relevance to the patient group, and familiarity of the clinicians with the issue they are faced with. Clinicians who often prescribe blood are seemingly less likely to take advice or change behaviours in response to computerised warnings.

Of note the majority of blood transfusions occur in HO and CC. Introduction of warnings has an effect on user prescribing in these areas, but this is not sustained. This may be linked to alert fatigue—outside CC and haematology where users do not encounter warnings very often, it is possible that they take more notice of them [29]. Considering that there is evidence on restricting transfusion in ICU, this is a relevant target for future studies to understand how CDS can create sustained behaviour change in this environment.

In CC overall the proportion of platelet transfusions given with a pre-transfusion platelet count > 10 × 10^9^/L fell during the study time but paradoxically the warnings appeared to transiently increase transfusions. In HO, platelet transfusions significantly fell in association with introduction of warnings. Since this is a largest area of use, clinician behaviour change here is important.

EHR can positively influence clinician behaviour but must be designed by the clinicians themselves and effects must be studied over time, as initial positive effects may wane. Where warnings are frequent, alert fatigue is likely and will not only result in warnings being ignored, but will frustrate and slow down clinicians. The EHR in our institution is designed in conjunction with the clinicians themselves and developed in an iterative manner. The software is built and controlled by the organisation so where clinicians do not approve of, or change their mind about, EHR design, the software is modified. Studies of this type check over time that behaviour changes are sustained. All EHR changes are accompanied by education and communication to users. The software build is safeguarded by clinicians involved in its management who have previously published on the effects of alert fatigue. This is important when institutions are considering CDS within EHR.

### Limitations

There are several limitations to this study. Importantly, the study includes all patients other than those in ED and those on a renal dialysis programme. Patients excluded from the 70 g/L threshold in national and local guidance, such as those who are chronically transfused, those with acute coronary syndrome, and those with major haemorrhage have been included in the data. Similarly patients undergoing procedures or who have active bleeding may receive appropriate platelet transfusions at thresholds higher than those given. No individual patient data were reviewed to examine whether transfusions were given at higher thresholds for valid clinical reasons. This would explain the seemingly very high proportion of patients who received a transfusion above accepted thresholds in our study. It is reasonable to assume that the proportion of patients receiving transfusions for those reasons would not change over time.

Although we have used a methodology that allowed us to analyse the longitudinal effect of the interventions [14], other confounding factors may have influenced RBC and platelet prescribing behaviour over the course of the study. Importantly, the introduction of Patient Blood Management, with the focus on evidence based practice and subsequent adoption of more restrictive transfusion thresholds worldwide and subsequent downward trends in blood use, may have had an impact on the prescribing practices of doctors in our institution, irrespective of alerts on the PICS [30]. In 2015 a dedicated transfusion consultant was appointed at our trust for the first time, and subsequently there has been more intensive clinical support for transfusion training and delivery.

## Conclusion

In summary, for blood transfusion, after introduction of EHR warnings, clinicians outside CC and HO prescribed fewer transfusions to patients with an haemoglobin of > 70 g/L than before the warnings were introduced. Platelet transfusions were reduced in critical care and haematology/oncology.

Appropriate use of warnings can reduce RBC and platelet transfusions to patients and this behaviour can be sustained. Warnings in electronic records must be used carefully to avoid fatigue. This is clinically important in terms of reduction of harm to patients themselves and reduction of poor use of precious resources.

## Supplementary Information

**Additional file 1: Table S1**: RBC transfusion models' parameters.** Table S2**: Platelet transfusion models' parameters.

## Data Availability

The extraction and analysis of data for the current study was done in-line with organisational policies; it is not publically available. An anonymised participant level dataset may be made available upon receipt of an application and necessary data sharing documentation being completed, please contact the corresponding author in the first instance.

## Abbreviations

CC: Critical care
CDS: Clinical decision support
CI: Confidence interval
ED: Emergency department
EHR: Electronic health records
HO: Heamatology/oncology
IQR: Interquartile range
PICS: Prescribing, Information and Communication System
RBC: Red blood cell
