# Supplementary material for: Effectiveness of clinical decision support in controlling inappropriate red blood cell and platelet transfusions, speciality specific responses and behavioural change
Source: BMC Med Inform Decis Mak. 2022 Dec 29;22:342. doi: 10.1186/s12911-022-02045-8 (PMC9798655; doi:10.1186/s12911-022-02045-8)
Supplement: Supplementary file 1 — Additional file 1: Table S1: RBC transfusion models' parameters. Table S2: Platelet transfusion models' parameters. [file 12911_2022_2045_MOESM1_ESM.docx]

**Effectiveness of clinical decision support in controlling inappropriate red blood cell and platelet transfusions**

Jolene Atia^1^, Felicity Evison^1^, Suzy Gallier^1,2^, Sophie Pettler^1,3^, Mark Garrick^1^, Simon Ball^1,4,5^, Will Lester^1^, Suzanne Morton^1,6^, Jamie Coleman^1,3^, Tanya Pankhurst^1^

^1^University Hospital Birmingham NHS Foundation Trust, Edgbaston, Birmingham, B15 2GW
^2^PIONEER: HDR-UK Health Data Research Hub for Acute Care, Institute of Inflammation and Ageing, University of Birmingham, Birmingham, B15 2GW
^3^School of Medicine, College of Medical and Dental Sciences, University of Birmingham, Edgbaston, Birmingham, B15 2TT, UK
^4^Health Data Research UK Midlands, University of Birmingham
^5^Institute of Infection and Immunity, University of Birmingham
^6^NHS Blood and Transplant, Vincent Drive, Edgbaston, Birmingham. B15 2SG

**Jolene Atia (PhD)***Intelligence Analyst, Department of Health Informatics*, University Hospitals Birmingham NHS Foundation Trust

**Felicity Evison (MSC)***Principal Research Informatician, Department of Health Informatics*, University Hospitals Birmingham NHS Foundation Trust

**Suzy Gallier (BSc)***Head of Informatics Research & Commercial Development,* University Hospitals Birmingham NHS Foundation Trust
*Head of Bio-informatics, Department of Health Informatics*, PIONEER: HDR-UK Health Data Research Hub in Acute care, University of Birmingham

**Sophie Pettler** **(MBChB)***Medical student*, School of Medicine, College of Medical Dental Sciences, University Birmingham
University Hospital Birmingham NHS Foundation, Edgbaston, Birmingham

**Mark Garrick (MSc)***Director of Strategy and Quality Development***,** University Hospital Birmingham NHS Foundation Trust, Edgbaston, Birmingham

**Simon Ball (MA, PhD, FRCP)***Executive Medical Director,* University Hospital Birmingham NHS Foundation Trust
HDRUK Better Care Science Priority and HDRUK Midlands
Institute of Infection and Immunity, University of Birmingham

**Will Lester (FRCP, FRCPath, PhD)***Consultant Haematologist,* University Hospital Birmingham NHS Foundation Trust

**Suzanne Morton (MRCP, FRCPath)***Consultant Haematologist***,** University Hospital Birmingham NHS Foundation Trust
NHS Blood and Transplant

**Jamie J Coleman (**MBChB, MA**, MD, FRCP)***Professor of Clinical Pharmacology and Medical Education/Consultant Physician and Associate Medical Director,* School of Medicine, College of Medical and Dental Sciences, University of Birmingham
University Hospital Birmingham NHS Foundation

**Tanya Pankhurst (MBBS, PhD, MRCP)***Consultant Nephrologist, Director of Digital Healthcare and Chief Clinical Information Officer,* University Hospital Birmingham NHS Foundation

**Supplementary Material**

| **Table S1: RBC transfusion models' parameters** | | | |  |  |  |  |  |  |  |  |  |
| --- | --- | --- | --- | --- | --- | --- | --- | --- | --- | --- | --- | --- |
|  | **Variables for non-CC/HO and HO models** | | | | | | | **Variables for CC model** | | | | |
| **Month** | **time (month)** | **Interv1** | **time after interv1** | **interv2** | **time after interv2** | **interv3** | **time after interv3** | **time (month)** | **Interv1** | **time after interv1** | **interv2** | **time after interv2** |
| Jan-10 | 1 | 0 | 0 | 0 | 0 | 0 | 0 | 1 | 0 | 0 | 0 | 0 |
| Feb-10 | 2 | 0 | 0 | 0 | 0 | 0 | 0 | 2 | 0 | 0 | 0 | 0 |
| Mar-10 | 3 | 0 | 0 | 0 | 0 | 0 | 0 | 3 | 0 | 0 | 0 | 0 |
| Apr-10 | 4 | 0 | 0 | 0 | 0 | 0 | 0 | 4 | 0 | 0 | 0 | 0 |
| May-10 | 5 | 0 | 0 | 0 | 0 | 0 | 0 | 5 | 0 | 0 | 0 | 0 |
| : : | : : | : : | : : | : : | : : | : : | : : | : : | : : | : : | : : | : : |
| Mar-12 | 27 | 0 | 0 | 0 | 0 | 0 | 0 | 27 | 0 | 0 | 0 | 0 |
| Apr-12 | 28 | 0 | 0 | 0 | 0 | 0 | 0 | 28 | 0 | 0 | 0 | 0 |
| May-12 | 29 | 0 | 0 | 0 | 0 | 0 | 0 | 29 | 0 | 0 | 0 | 0 |
| Jun-12 | 30 | 1 | 1 | 0 | 0 | 0 | 0 | 30 | 1 | 1 | 0 | 0 |
| Jul-12 | 31 | 1 | 2 | 0 | 0 | 0 | 0 | 31 | 1 | 2 | 0 | 0 |
| Aug-12 | 32 | 1 | 3 | 0 | 0 | 0 | 0 | 32 | 1 | 3 | 0 | 0 |
| Sep-12 | 33 | 1 | 4 | 0 | 0 | 0 | 0 | 33 | 1 | 4 | 0 | 0 |
| Oct-12 | 34 | 1 | 5 | 0 | 0 | 0 | 0 | 34 | 1 | 5 | 0 | 0 |
| : : | : : | : : | : : | : : | : : | : : | : : | : : | : : | : : | : : | : : |
| Mar-15 | 63 | 1 | 34 | 0 | 0 | 0 | 0 | 63 | 1 | 34 | 0 | 0 |
| Apr-15 | 64 | 1 | 35 | 0 | 0 | 0 | 0 | 64 | 1 | 35 | 0 | 0 |
| May-15 | 65 | 1 | 36 | 1 | 1 | 0 | 0 | 65 | 1 | 36 | 1 | 1 |
| Jun-15 | 66 | 1 | 37 | 1 | 2 | 0 | 0 | 66 | 1 | 37 | 1 | 2 |
| Jul-15 | 67 | 1 | 38 | 1 | 3 | 0 | 0 | 67 | 1 | 38 | 1 | 3 |
| Aug-15 | 68 | 1 | 39 | 1 | 4 | 0 | 0 | 68 | 1 | 39 | 1 | 4 |
| Sep-15 | 69 | 1 | 40 | 1 | 5 | 0 | 0 | 69 | 1 | 40 | 1 | 5 |
| Oct-15 | 70 | 1 | 41 | 1 | 6 | 0 | 0 | 70 | 1 | 41 | 1 | 6 |
| Nov-15 | 71 | 1 | 42 | 1 | 7 | 0 | 0 | 71 | 1 | 42 | 1 | 7 |
| Dec-15 | 72 | 1 | 43 | 1 | 8 | 0 | 0 | 72 | 1 | 43 | 1 | 8 |
| Jan-16 | 73 | 1 | 44 | 1 | 9 | 0 | 0 | 73 | 1 | 44 | 1 | 9 |
| Feb-16 | 74 | 1 | 45 | 1 | 10 | 0 | 0 | 74 | 1 | 45 | 1 | 10 |
| Mar-16 | 75 | 1 | 46 | 1 | 11 | 0 | 0 | 75 | 1 | 46 | 1 | 11 |
| Apr-16 | 76 | 1 | 47 | 1 | 12 | 0 | 0 | 76 | 1 | 47 | 1 | 12 |
| May-16 | 77 | 1 | 48 | 1 | 13 | 0 | 0 | 77 | 1 | 48 | 1 | 13 |
| Jun-16 | 78 | 1 | 49 | 1 | 14 | 0 | 0 | 78 | 1 | 49 | 1 | 14 |
| Jul-16 | 79 | 1 | 50 | 0 | 0 | 1 | 1 | 79 | 1 | 50 | 1 | 15 |
| Aug-16 | 80 | 1 | 51 | 0 | 0 | 1 | 2 | 80 | 1 | 51 | 1 | 16 |
| Sep-16 | 81 | 1 | 52 | 0 | 0 | 1 | 3 | 81 | 1 | 52 | 1 | 17 |
| Oct-16 | 82 | 1 | 53 | 0 | 0 | 1 | 4 | 82 | 1 | 53 | 1 | 18 |
| Nov-16 | 83 | 1 | 54 | 0 | 0 | 1 | 5 | 83 | 1 | 54 | 1 | 19 |
| : : | : : | : : | : : | : : | : : | : : | : : | : : | : : | : : | : : | : : |
| Jul-19 | 115 | 1 | 86 | 0 | 0 | 1 | 37 | 115 | 1 | 86 | 1 | 51 |
| Aug-19 | 116 | 1 | 87 | 0 | 0 | 1 | 38 | 116 | 1 | 87 | 1 | 52 |

Password warnings for RBC transfusion: (1)Interv1 in May 2012: RBC prescription warning for patients who had a haemoglobin ≥ 100 g/L. (2)Interv2 in May 2015: (in addition to the first warning): in non-CC patients if haemoglobin was ≥80 g/L and in CC if haemoglobin was ≥70 g/L in response to prescription of RBCs. (3) Interv3 in July 2016: (replacing the warning introduced in May 2015) triggered for all patients if haemoglobin was ≥70 g/L in all clinical locations.

| **Table S2: Platelet transfusion models' parameters** | | | |  |  |
| --- | --- | --- | --- | --- | --- |
|  | **Variables for non-CC/HO, CC and HO models** | | | | |
| **Month** | **time (month)** | **Interv 1** | **time after interv1** | **interv2** | **time after interv2** |
| Jan-10 | 1 | 0 | 0 | 0 | 0 |
| Feb-10 | 2 | 0 | 0 | 0 | 0 |
| Mar-10 | 3 | 0 | 0 | 0 | 0 |
| Apr-10 | 4 | 0 | 0 | 0 | 0 |
| May-10 | 5 | 0 | 0 | 0 | 0 |
| : : | : : | : : | : : | : : | : : |
| Mar-15 | 63 | 0 | 0 | 0 | 0 |
| Apr-15 | 64 | 1 | 1 | 0 | 0 |
| May-15 | 65 | 1 | 2 | 0 | 0 |
| Jun-15 | 66 | 1 | 3 | 0 | 0 |
| Jul-15 | 67 | 1 | 4 | 0 | 0 |
| Aug-15 | 68 | 1 | 5 | 0 | 0 |
| Sep-15 | 69 | 1 | 6 | 0 | 0 |
| Oct-15 | 70 | 1 | 7 | 0 | 0 |
| Nov-15 | 71 | 1 | 8 | 0 | 0 |
| Dec-15 | 72 | 1 | 9 | 0 | 0 |
| Jan-16 | 73 | 1 | 10 | 0 | 0 |
| Feb-16 | 74 | 1 | 11 | 0 | 0 |
| Mar-16 | 75 | 1 | 12 | 0 | 0 |
| Apr-16 | 76 | 1 | 13 | 0 | 0 |
| May-16 | 77 | 1 | 14 | 0 | 0 |
| Jun-16 | 78 | 1 | 15 | 1 | 1 |
| Jul-16 | 79 | 1 | 16 | 1 | 2 |
| Aug-16 | 80 | 1 | 17 | 1 | 3 |
| Sep-16 | 81 | 1 | 18 | 1 | 4 |
| Oct-16 | 82 | 1 | 19 | 1 | 5 |
| Nov-16 | 83 | 1 | 20 | 1 | 6 |
| : : | : : | : : | : : | : : | : : |
| Jul-19 | 115 | 1 | 52 | 1 | 38 |
| Aug-19 | 116 | 1 | 53 | 1 | 39 |

Password warnings for platelets transfusion: (1)Interv 1 in May 2015: platelets prescription warning for patients who had a platelet count > 20x10^9^ /L. (2)Interv 2 in July 2016: limit lowered to platelet count >10x10^9^ /L.
